# Near-Plasma Chemical Surface Engineering

**DOI:** 10.3390/nano14020195

**Published:** 2024-01-15

**Authors:** Paula Navascués, Urs Schütz, Barbara Hanselmann, Dirk Hegemann

**Affiliations:** Laboratory for Advanced Fibers, Empa, Swiss Federal Laboratories for Materials Science and Technology, Lerchenfeldstrasse 5, 9014 St. Gallen, Switzerland

**Keywords:** low-pressure plasma, plasma etching, plasma polymerization, surface reactions, wettability, porous SiOx

## Abstract

As a new trend in plasma surface engineering, plasma conditions that allow more-defined chemical reactions at the surface are being increasingly investigated. This is achieved by avoiding high energy deposition via ion bombardment during direct plasma exposure (DPE) causing destruction, densification, and a broad variety of chemical reactions. In this work, a novel approach is introduced by placing a polymer mesh with large open area close to the plasma–sheath boundary above the plasma-treated sample, thus enabling near-plasma chemistry (NPC). The mesh size effectively extracts ions, while reactive neutrals, electrons, and photons still reach the sample surface. The beneficial impact of this on the plasma activation of poly (tetrafluoroethylene) (PTFE) to enhance wettability and on the plasma polymerization of siloxanes, combined with the etching of residual hydrocarbons to obtain highly porous SiOx coatings at low temperatures, is discussed. Characterization of the treated samples indicates a predominant chemical modification yielding enhanced film structures and durability.

## 1. Introduction

Low-pressure plasma technology attracts attention as a versatile and powerful surface engineering approach. A huge variety of plasma sources and operating conditions, consequence of decades-long development, provide the basis to precisely modify surface properties by depositing materials and, generally, tuning surfaces at the nanoscale [1,2]. Non-thermal low-pressure plasma technology is, as stated by these attributes, able to carry out processes at room temperature thanks to the highly non-equilibrium conditions achieved by applying an electromagnetic field to a gas at reduced pressure conditions [3]. Taking into account the challenges that humanity is currently facing, plasma surface engineering stands out because of its solventless nature, the reduced production of byproducts, its operation by electricity, and its potential for scaling-up, for example, with roll-to-roll designs [4,5,6].

Plasma surface engineering covers different approaches from surface activation and etching to the deposition of uniform thin films and a plethora of nanostructures [7]. These processes end up in specific control of the surface properties without affecting those of the bulk material [1]. Therefore, the kind of plasma process performed relies on the purpose or application of the material being treated. Correspondingly, the plasma operating conditions might be very different, for example, if the goal is to fabricate a dense barrier coating, or a porous thin film. In this regard, the methodology presented in this article focuses on two topics of high interest nowadays: the enduring control of surface wettability [8,9,10], and the fabrication of inherently nanoporous materials for various functional applications [11,12,13].

In recent studies of plasma polymerization applied to complex geometries, it has been observed that the zones of the substrate that were not directly exposed to the plasma were characterized by a different chemical composition [14,15]. Specifically, by studying the polymerization of hexamethyldisiloxane (HMDSO), as well as etching experiments with O_2_ in the plasma, the relevance of plasma chemical reactions has been highlighted, which take place at surfaces shielded from the plasma [14]. Due to the fact that these regions are not directly exposed to the discharge, they are not affected by the bombardment of high-energy heavy particles—mainly positive ions—allowing the diffusing species to define the surface reactions. Moreover, it is known that, to deposit nanoporous plasma polymer films (PPFs) from HMDSO, rather mild plasma operating conditions are required, limiting the influence of high-energy particles and, therefore, crosslinking and densification [16]. Likewise, an adjusted plasma activation of polymers, opening chains and promoting specific reactions at the surface, supports the durability of surface wettability [9].

To avoid the influence of high-energy plasma components, remote configurations [17,18]—sometimes also reported as downstream configurations [19,20] or afterglows [21]—have been studied for plasma deposition and etching. In these plasma reactor configurations, the substrate is placed outside the active plasma zone at a distance to allow radicals produced in the plasma to reach, at least partially, the surface [22]. These approaches benefit from the plasma production of radicals, dealing with the fabrication [23] or modification [24] of nanostructures in a mild environment. However, due to its afterglow nature, only those long-living plasma species characterized by large mean free paths likely play a role in this process, highly decreasing its deposition [25] and etching rates [26]. Therefore, parts of the benefits of plasma surface engineering are lost. In some configurations, mainly using inductively coupled plasma (ICP), a grid is introduced to separate the plasma source from a second region where the plasma can diffuse by passing the (biased) grid. This grid biasing method allows to enhance the plasma etching and deposition specificity in the second region by lowering the electron temperature due to inelastic gas-phase collisions [27,28]. In all configurations, the considered zones extend to several centimeters, i.e., the substrate is placed far from the primary plasma zone, reducing its interaction with reactive species.

**Figure 1:**
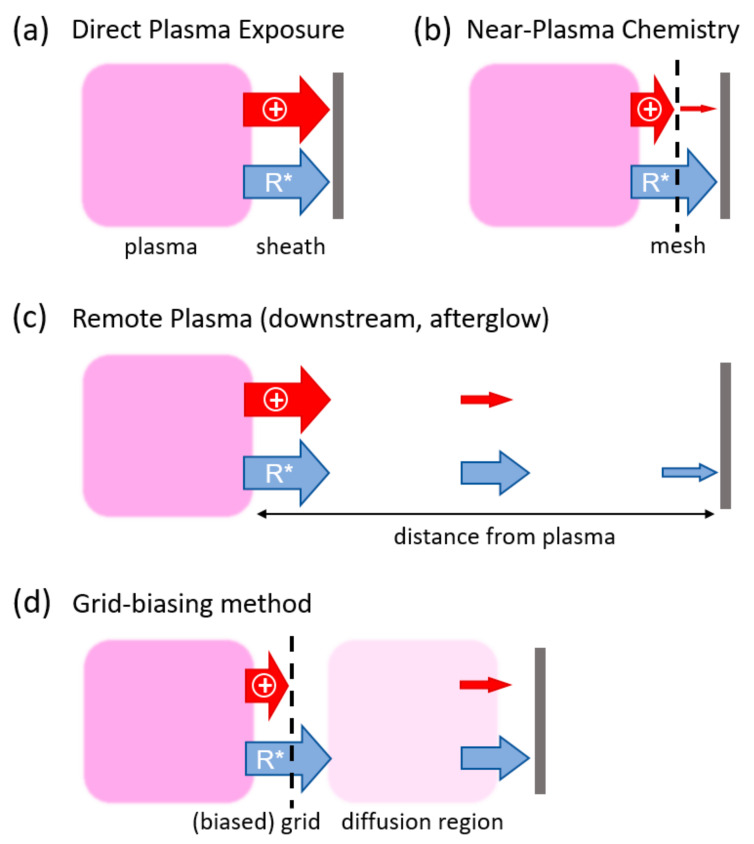
Schematic drawing of different forms of plasma–substrate interactions. (**a**) Direct plasma exposure; (**b**) near-plasma chemistry with mesh; (**c**) remote plasma; and (**d**) grid biasing method with plasma diffusing through the grid. The widths of each arrow indicate the contribution of reactive species (R*) and energetic particles (+) towards the position of the substrate. NPC, introducing a mesh between the plasma and the substrate, effectively blocks ions from reaching the substrate, while reactive species can diffuse.

To overcome such limitations, in this study, a new methodology is introduced, referred to as “near-plasma chemistry” (NPC). By using a polymeric mesh located close to the substrate being treated, the influence of high-energy species is strongly reduced, while electrons, photons, and neutrals can still pass through. This approach has been applied to two different room-temperature plasma processes at low pressure. On the one hand, the activation of poly (tetrafluoroethylene) (PTFE) surfaces to enhance wettability has been considered. On the other hand, cycles of polymerization and etching alternately using Ar/O_2_/HMDSO and Ar/O_2_ plasmas to obtain highly porous SiOx coatings have been further optimized [29]. In contrast to other strategies for lowering the effect of energetic particles, in the presented approach, as portrayed in Figure 1, the mesh is located just millimeters above the sample. The substrate is thus as close to the plasma region as it is in direct plasma exposure (DPE), while no plasma is ignited between the mesh and the substrate. The obtained results, which show enhanced wettability and porosity, indicate that the introduced NPC surface engineering provides highly defined plasma chemical modifications at the surface, offering a promising methodology for the precise tuning of surface properties at room temperature.

There has long been interest in increasing the wettability of PTFE, which is an inert polymer exhibiting water- and oil-repellant properties, thus hampering, for example, adhesion processes [30,31]. Plasma activation has been recognized to induce defluorination, oxygen incorporation, and changes in morphology [32,33]. Aging processes, however, result in surface reorientation of the modified PTFE via thermodynamic relaxation, yielding so-called hydrophobic recovery [34,35]. Different plasma processing methods and gas compositions have thus been investigated to attenuate a hydrophobic recovery that typically yields an almost complete loss of hydrophilicity on PTFE within several days [31,36,37]. Starting from an optimized gas composition, as reported in the literature using an inert gas (Ar or He) mixed with NH_3_/H_2_O [38,39], it is demonstrated that NPC further enhances the durability of wettable PTFE surfaces.

Considering the plasma deposition of functional materials, porous PPFs can be obtained from the combination of HMDSO plasma polymerization and etching processes [29,40]. In the present study, the NPC approach is applied to these alternating plasma processes to increase the porosity of SiOx coatings. By optimizing the plasma operating parameters through applying the NPC strategy, a porous volume as high as 23% has been achieved. These values are remarkable considering the context of room-temperature plasma processing, close to the maximum that might be obtained using this approach according to simulations of the Si-O network [29].

## 2. Materials and Methods

**Figure 2:**
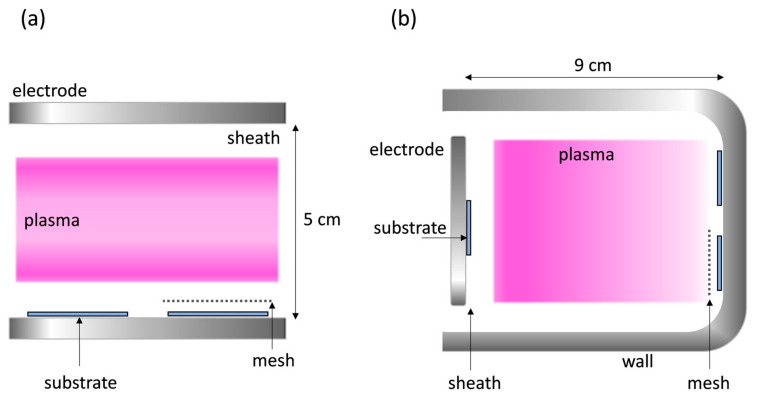
Schematic representation of the experimental configurations. (**a**) Symmetric reactor used for plasma activation of PTFE and (**b**) asymmetric reactor for the fabrication of porous SiOx coatings.

The experiments were performed in two capacitive coupled plasma (CCP) reactors driven by radiofrequency (RF, 13.56 MHz) with different geometries, referred to as symmetric and asymmetric configurations. Briefly, the symmetric set-up (see Figure 2a) consists of two plane-parallel electrodes with 30 cm diameters, separated by 5 cm [14,41]. The asymmetric reactor configuration (see Figure 2b) comprises a driven electrode with an area of 21 × 70 cm^2^, separated 9 cm from the chamber walls [29,41]. A gas showerhead has been used in both cases to ensure uniform gas flow conditions through the plasma zone towards the substrates. In the symmetric reactor, substrates were placed on the bottom electrode directly exposed to the plasma, separated only by the plasma sheath. Due to the absence of a bias potential, the substrate temperature remained close to room temperature. In the asymmetric configuration, substrates were mounted both at the RF electrode and at the grounded reactor walls, as indicated in Figure 2. In the latter case, the substrates are less exposed to ion bombardment, enabling room-temperature conditions, since the plasma density is highest close to the electrode and steadily reduces towards the wall. Nonetheless, the samples are in contact to the plasma, separated by a larger sheath in front of the RF electrode and a thin sheath close to the wall. Hence, the configurations used also differ in their respective electron energy distribution functions [42].

To study the effect of near-plasma chemistry, a polymeric mesh was introduced between the substrate and the bulk plasma to avoid direct plasma exposure by maintaining the same conditions otherwise. Therefore, in addition to the experiments fully exposed to the plasma, substrates were covered by a mesh located 4 mm above the sample, that is, placed near the plasma–sheath boundary (or in the plasma sheath). Hence, no plasma is present between the mesh and the sample. The mesh is made of polymeric fibers (polyamide PA 6.6) 40 µm thick (therefore acting as an electric insulator) and separated by 240 µm, leaving an open area of 78%. The mesh number (number of fibers per inch) is thus 87.5.

Two different sets of experiments were conducted using the two different CCP reactors. The experiments were conducted more than three times, under the same conditions or with slight variations, to ensure reproducibility. On the one hand, plasma activation of thin poly (tetrafluoroethylene) (PTFE) films was carried out in the symmetric reactor with Ar/NH_3_/H_2_O gaseous mixtures (20/20/2 sccm gas flow rates). Input power and pressure were fixed to 30 W (22 W absorbed power) and 7 Pa, respectively. The treatment time was 15 min. Substrates fully exposed to the plasma or covered by the mesh were compared.

The second set of experiments was performed in the asymmetric reactor. Cycles of plasma polymerization and etching experiments were conducted, as previously studied, and optimized to fabricate nanoporous SiOx PPFs [29]. The monomer used was hexamethyldisiloxane (HMDSO, Sigma Aldrich, Buchs, Switzerland) with Ar/O_2_ added for plasma polymerization, while Ar/O_2_ plasma without monomer was used for etching purposes. The monomer flow rate varied between 4, 5, and 6 sccm, while the flow rates of Ar/O_2_ were set at 20/40 sccm. Prior to the plasma polymerization experiments, the substrates were cleaned with Ar/O_2_ plasma. Each cycle of plasma polymerization lasted 3.5 min, while 5 min were used for the intermittent etching. The operating pressure was set at 7 Pa for all processes, while the plasma input power was varied. The absorbed power was approximately half of the input (i.e., forward RF) power. Experiments with the substrates directly exposed to the plasma were compared to those with the mesh installed for all process steps (plasma cleaning, deposition, and etching).

For the PTFE activation experiments, water contact angles (WCAs) were measured in static mode with 2 µL drops (DSA25, Krüss, Hamburg, Germany). Initial measurements were carried out within 1–2 h after treatment, ensuring that the surfaces were stable. The samples were stored in an air-conditioned environment (23 °C, 50% relative humidity) to follow aging over time. The etching rate was calculated from weighing the PTFE films (7 × 14 cm^2^) before and after plasma etching using a microbalance (Mettler Toledo XS204, 0.1 mg precision), applying charge neutralization. Chemical characterization was performed via ATR-FTIR spectroscopy (Varian 640-IR, Agilent Technologies, Santa Clara, CA, USA, 4 cm^−1^ resolution, 64 scans) pressing the plasma-treated PTFE directly against the ATR crystal. Changes in morphology were examined via scanning electron microscopy (SEM) using a Hitachi S-4800 microscope (Hitachi, Tokyo, Japan) at 2 kV, detecting secondary electrons at a high magnification. Before introducing the samples into the microscope, they were sputter-coated with Au/Pd to provide sufficient electrical conductivity. For the fabrication of SiOx porous coatings in the symmetric reactor, silicon wafers (p-type) and aluminum (Al) foils were used as substrates, depending on the characterization technique. The thickness of the PPFs was assessed on Si wafers using profilometry (Dektak XT, Bruker France, Palaiseau, France), while Al foil was selected for ATR-FTIR spectroscopy. Moreover, ellipsometry measurements were carried out for the PPFs deposited on the Si wafers, using an ellipsometer (Nanofilm EP4, Accurion, Göttingen, Germany) operating with a constant wavelength of 658 nm and varying the angle of incidence. Porosity values were obtained from the refractive index (determined modeling with a Cauchy dispersion module), applying the volume averaging theory (VAT) with an effective medium approximation. More information about ellipsometry measurements, modeling, and porosity assessments can be found in a previous publication [29].

## 3. Results

Here, the impact of near-plasma chemistry (NPC) through the introduction of a polymeric mesh between the plasma and the substrate is compared to the common direct plasma exposure (DPE) method for a plasma-treated substrate, that is, using exactly the same plasma conditions, with and without the mesh. Therefore, all gas-phase processes remain undisturbed independent of the configuration, with or without mesh.

### 3.1. Activation of PTFE for Wettability Control

**Figure 3:**
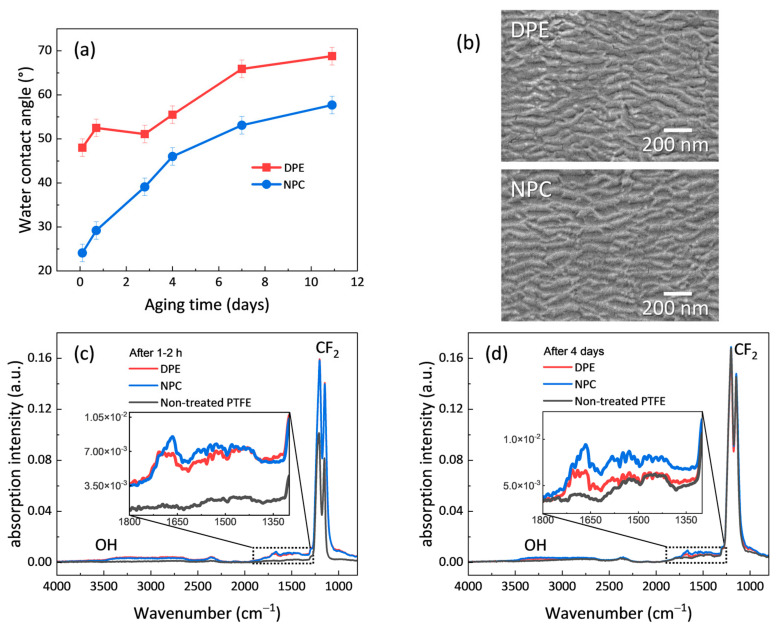
Comparison of plasma-treated PTFE under DPE and NPC conditions (15 min, Ar/NH_3_/H_2_O plasma). (**a**) WCA measured over time, stored at 23 °C and 50% RH. (**b**) SEM images of plasma-treated PTFE surfaces (top: DPE vs. bottom: NPC). ATR-FTIR of plasma-treated PTFE, recorded (**c**) 1–2 h and (**d**) 4 days after plasma treatment, compared to non-treated PTFE.

The first experiment performed to demonstrate the potential of NPC considers the challenging plasma activation of PTFE substrates. As is known from the literature, the combined action of NH, H, and OH species in Ar/NH_3_/H_2_O plasma enables defluorination and the introduction of polar groups, revealing delayed aging processes [38]. From this, the operating conditions have been adjusted and optimized regarding the reactor geometry used here. Under DPE conditions, the originally hydrophobic PTFE surface with a water contact angle (WCA) of 118° remains rather hydrophilic (WCA < 70°) over more than one week of storage in an air-conditioned environment after plasma treatment. As seen from Figure 3, using NPC with a mesh further enhances the wettability of PTFE, showing attenuated aging behavior, and leaving a WCA still below 60° after 11 days. The observed plasma etching rates were 5.0 ± 0.4 nm min^−1^ for DPE and 3.1 ± 0.4 nm min^−1^ for NPC. Taking into account the reduced flux of reactive species to the substrate due to the mesh interaction, the etching rate is only slightly reduced for NPC compared to the ion-assisted DPE etching. Accordingly, after 15 min of plasma exposure, the same etching structures were detected with SEM for both conditions (Figure 3b). Note that the pristine PTFE surface has a smooth appearance showing no particular features (thus not shown here). Importantly, the observed NPC-induced morphology did not reveal any hints of a pattern that might be caused by shadowing effects of the mesh.

To indicate the plasma chemical modification of PTFE in both configurations, ATR-FTIR spectra were recorded within 1–2 h after plasma activation, agreeing with the first measurement of the WCA upon aging. All spectra are dominated by the CF_2_ bands of PTFE at 1150 cm^−1^ and 1204 cm^−1^, which can be attributed to the asymmetric and the symmetric stretching of the CF_2_ groups, respectively. These bands appear to be broadened after plasma treatment, indicating a slightly altered chemical environment due to chain scission, defluorination, the formation of radical sites, and related transformation of CF_2_ groups, which might also involve UV radiation [43]. A noticeable chemical modification becomes visible with the increase in the broadband at 3000–3500 cm^−1^, according to the stretching of OH groups, and in the range of 1300–1750 cm^−1^, indicating the incorporation of O and probably also N functional groups (C=O, COOH, COO^−^ and CNH groups) [34,36]. NPC compared to DPE shortly after plasma treatment, however, only yields a slightly increased functionality, as observed around 1675 cm^−1^. More differences become visible after 4 days of aging. On the one hand, the broadening of the CF_2_ bands has largely disappeared, indicating the relaxation and reorientation of CF_2_ groups. On the other hand, functional groups can still be observed for NPC conditions, while they are diminished for DPE, agreeing with the trend in WCA during aging.

### 3.2. Cyclic Polymerization and Etching of HMDSO Coatings

**Figure 4:**
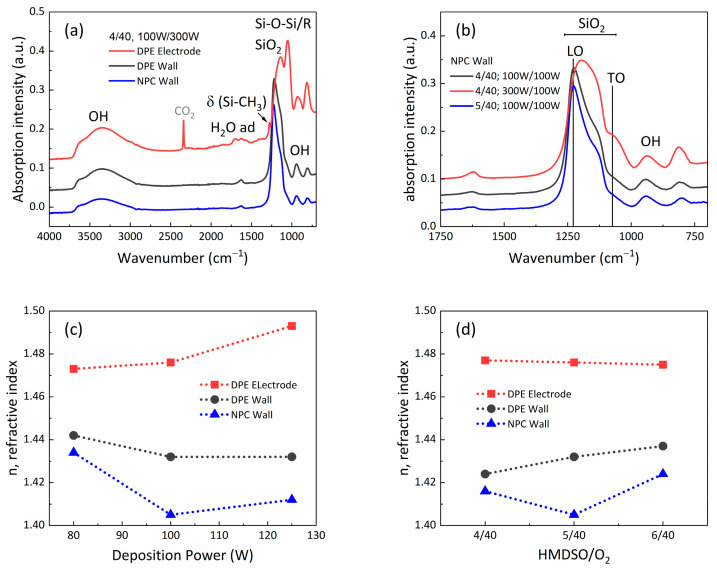
Deposition of porous SiOx coatings in the asymmetric configuration. ATR-FTIR spectra for samples fabricated (**a**) at different positions in the reactor (4:40 HMDSO/O_2_ ratio, 100 W deposition, 300 W etching) and (**b**) at the wall, applying NPC for different operating conditions. Evolution of the refractive index of the porous coatings as a function of (**c**) the deposition power (4:40 HMDSO/O_2_ ratio and etching at 100 W) and as a function of (**d**) the HMDSO/O_2_ ratio (deposition and etching at 100 W).

The fabrication of porous SiOx coatings was carried out in the asymmetric reactor. The substrates were placed at the reactor wall to reduce the influence of high-energy particles during film growth. The methodology used consisted of cycles of plasma polymerization of Ar/O_2_/HMDSO and subsequent etching with Ar/O_2_ plasmas to remove residual hydrocarbons. The resulting material consisted of an open Si–O–Si network comprising interconnected nanopores with Si-OH functionalized pore walls [29]. For comparative purposes, a substrate directly exposed to the plasma (DPE) was also located at the RF electrode. Figure 4a,b show the characteristic ATR-FTIR spectra of plasma polymer films (PPFs) fabricated at different positions and with varied operating parameters. The main bands detected can be assigned to the stretching vibrations of SiO_2_ at 1074 cm^−1^ and 1228 cm^−1^, corresponding to its transverse optical (TO) and longitudinal optical (LO) vibration modes [44]. Furthermore, the main band related to OH groups is detected in the region between 3000 and 3500 cm^−1^. Accordingly, the silanol (Si-OH) characteristic vibration is detected around 930 cm^−1^, as well as that of adsorbed H_2_O, at around 1632 cm^−1^ [29]. For the spectrum of the reference coating at the RF electrode (see the red curve in Figure 4a), the δ (Si-CH_3_) band is faintly detected at 1259 cm^−1^, related to a residual (non-etched) content of hydrocarbons from the polymerization of HMDSO [45]. Due to the higher deposition rate at the electrode, not all hydrocarbons were removed during the etching cycle while conditions were optimized to allow complete oxidation at the wall. Note that a uniform deposition profile has also been obtained for NPC using the mesh.

Figure 4a shows three curves plotted for the different sample locations: DPE at the electrode and at the wall, as well as at the wall covered by the mesh (i.e., NPC). It can be observed that the spectrum corresponding to the NPC at the wall is characterized by a narrowing of the SiO_2_ vibration, shifted to the LO mode. Similarly, this shifting is enhanced when using a 5:40 HMDSO/O_2_ ratio, compared to other operating conditions with the mesh at the wall, as shown in Figure 4b.

Figure 4c,d depict the evolution of the refractive index, determined using ellipsometry, as a function of the (c) etching power and (d) HMDSO/O_2_ ratio for samples at the electrode (DPE) and at the wall, without (DPE) and with the mesh (NPC). It can be observed that, for all cases, the samples subject to NPC are characterized by the lowest refractive index values. Moreover, the different operating parameters indicate that the minimum refractive index is obtained for the 5:40 HMDSO/O_2_ ratio for 100 W of power applied in the deposition and etching steps. This refractive index value of 1.405 corresponds to a volumetric porosity of 23%, using the volume averaging theory (VAT) with an effective medium approximation. Table 1 shows the refractive index (and porosity) values obtained for the different plasma operating parameters as varied for PPFs fabricated at the wall via NPC surface engineering. More details related to these findings and the tendencies shown in Figure 4 and Table 1 are discussed in the next section.

## 4. Discussion

### 4.1. Characteristics of Near-Plasma Chemistry

Introducing a mesh in between the plasma and the sample modifies the way the plasma interacts with the sample, whereas the bulk plasma remains unaffected. Since the mesh is placed in the plasma sheath region, it acquires a net negative potential with respect to the plasma potential (but still positive compared to the wall or electrode). In the asymmetric case with the mesh placed 4 mm above the wall (counter electrode), the mesh is close to the plasma–sheath boundary and might thus be considered to be at floating potential:

(1) $$∆\phi =\frac{T_{e}}{2e}ln\left(0.433\frac{m_{i}}{m_{e}}\right),$$

with electron temperature *T_e_* (in eV), ion mass *m_i_*, and electron mass *m_e_*. For the considered conditions with *T_e_* ≈ 2.3 eV and prevailing Ar ions [41], it follows that the mesh acquires a negative potential of about 12 V relative to the plasma. Hence, positive-charged ions are attracted by the mesh. Following the discussion by He et al. [46], the penetration depth of this potential into the plasma around the fibers of the mesh can be estimated as follows:

(2) $$d=1.02{\left(\sqrt{\frac{e∆\phi }{T_{e}}}-\frac{1}{\sqrt{2}}\right)}^{\frac{1}{2}}\left(\sqrt{\frac{e∆\phi }{T_{e}}}+\sqrt{2}\right)\lambda_{D},$$

with the Debye length *λ_D_* (in cm):

(3) $$\lambda_{D}\approx 743{\left(\frac{T_{e}\left[eV\right]}{n_{e}\left[{cm}^{-3}\right]}\right)}^{\frac{1}{2}},$$

considering the electron density *n_e_*. Note that Equations (1)–(3) rely on certain assumptions and approximations and should thus be used more as a guidance to select a suitable mesh size for different plasma operation conditions. Again, in the asymmetric case with the conditions used here, *n_e_* can be assumed to be around 2∙10^9^ cm^−3^ [41], and *λ_D_* ≈ 0.25 mm. Applying Equation (2), the penetration depth becomes around 3.6 mm, that is, much larger than the mesh size of 0.24 mm. Hence, ions (and charged particles such as dust) are effectively extracted and do not reach the sample underneath the mesh. Note that the mesh number (number of fibers per inch) could thus be further reduced to enlarge the open area. It should be mentioned that charge transfer collisions generate fast neutrals that still pass through the mesh [14]. Their flux and energy, however, is strongly limited when using the mesh in front of the grounded wall in the asymmetric set-up, since it attracts ions directly by entering the plasma sheath [41]. Hence, energy deposition by the energetic particles and plasma-related physical effects such as densification at the sample surface can be avoided. The situation is similar in the symmetric reactor. The immersion of the mesh within the plasma sheath yields a shift in the potential difference between the plasma bulk and mesh to values exceeding the floating potential [47], while lower *T_e_* and higher *n_e_*, as observed in symmetric plasmas, reduce *λ_D_* [41,42]. Considering these effects, the penetration depth is still much larger than the mesh size and ions are effectively extracted. It should be noted, however, that charge transfer contributes to the generation of fast neutrals, albeit with limited energy depending on the distance of the mesh to the plasma–sheath boundary.

Furthermore, the role of electrons should be considered. RF plasmas have a window during one RF cycle to allow fast electrons to reach the electrodes and walls. As the mesh potential lies between plasma potential and electrode/wall potential and electrons are much faster than ions, only those of the fast electrons directly hitting the fibers of the mesh are extracted [27,28]. Hence, a large portion of the fast electrons can reach the sample underneath the mesh. The same holds for neutral species such as radicals that can pass the mesh as well as VUV radiation. Near-plasma chemistry is thus distinguished by its proximity to the active plasma zone as in direct plasma treatment, but largely avoids energy deposition by heavy particles. This plasma-related surface engineering thus uses chemically reactive neutrals, photons, and electrons (with energies above bond energies), allowing us to perform a more-defined surface chemistry for deposition and etching processes. Note that the surplus of arriving negative charge carriers, that is, the electrons, might contribute to surface charging but also induce processes such as secondary electron emission to allow charge balancing. Since no shadowing effect of the mesh has been observed, it can be assumed that the electrons arrive with a sufficient angle distribution to allow uniform NPC surface engineering.

### 4.2. Impact of Near-Plasma Chemistry on Plasma Etching

Milder plasma conditions have been identified to reduce the aging of PTFE, since the action of both radicals and ions yield increased surface destruction effects [34]. It can thus be postulated that NPC results in less destructive conditions at the substrate surface, while a similar yet more-defined chemistry is promoted, supported by electron instead of ion interactions. Indeed, it has recently been discussed that electron interactions during plasma chemical etching in a remote plasma without ion bombardment can reduce surface damage, including atom displacement, surface roughness, and the removal of undesired materials [26]. Furthermore, the influence of trapped charges should be considered for aging effects [35]. It has been reported that accelerated ions hitting a polymer surface can become trapped at defect sites within the near-surface polymer region [48]. Trapped charge carriers, both positive and negative, i.e., including electrons, might contribute to the thermodynamic relaxation of plasma-activated PTFE surfaces through their decay over time—beside the mobility of fragments formed by the plasma interaction and the structural rearrangement of macromolecules [35]. The observed attenuated hydrophobic recovery might thus be related to the reduced NPC destruction effects and charge trapping (limited to electrons) by avoiding ion bombardment. Moreover, the broadening of CF_2_ bands in the ATR-FTIR spectrum indicates the presence of plasma-induced defect sites where charges might become trapped. Therefore, the relaxation of the network is accompanied by hydrophobic recovery, less pronounced for NPC, since more functional groups are still visible that agree with the WCA measurements.

According to the SEM images, the etching structures appear to be similar for both DPE and NPC, also agreeing with etching structures reported for Ar/NH_3_/H_2_O plasmas under different conditions [38]. Likewise, the observed etching rate for NPC, taking into account the reduced flux of reactive species to the substrate due to the interaction of the mesh, reflects a minor reduction in the etching effect compared to ion-assisted plasma etching. Hence, the overall gas flow dynamics might not be strongly affected by the mesh regarding diffusion of the reactive species from the bulk plasma [29,41]. In addition, it should be considered that VUV radiation also contributes to chain scission and depletion of fluorine from the PTFE surface, where its efficiency has been found to be further increased by the synergistic effects of hydrogen atoms [30,31,49]. It can thus be stated that NPC etching, avoiding ions yet allowing radicals, electrons, and photons to reach the substrate surface, yields a more-defined plasma chemical interaction, reducing substrate damaging, while maintaining efficient etching.

### 4.3. Impact of Near-Plasma Chemistry on Deposition/Etching Processes

For the deposition of porous SiOx coatings, the effect of reducing ion bombardment can already be observed in common DPE conditions when placing the sample at the reactor wall instead of at the electrode. A shift to higher wavelengths in ATR-FTIR, in particular to the LO vibration mode of SiO_2_, has been systematically detected, and is even stronger and narrowed when the NPC approach is used at the wall. This finding might indicate the formation of a defined Si–O–Si cage structure [50]. Accordingly, for all varied parameters, a correlation between this shift and the increase in porosity (i.e., a decrease in the refractive index) has been found. It is thus proposed that the partial or total elimination of ion-induced effects avoids the collapse and densification by crosslinking of the Si-O network, leading to higher porosities. Specifically, with the asymmetric reactor configuration, a maximum porous volume of 23% has been found for deposition and etching powers of 100 W using an HMDSO/O_2_ ratio of 5:40. This value is the highest reported, to the best of our knowledge, for this kind of system—porous SiOx—fabricated by plasma polymerization of HMDSO at room temperature. Typically, enhanced temperatures (250–400 °C) and post-treatment is required to achieve a similar porosity using DPE [51]. The application of NPC surface engineering, on the contrary, with consequent more-defined chemical reactions at the surface, allows for the deposition of a stable, open Si–O–Si network as well as an effective etching of residual hydrocarbons to produce nanoporous coatings. As for the plasma etching of polymers, VUV radiation plays an important role in the abstraction of residual hydrocarbons from the SiOCH network [20,52]. NPC is thus characterized by radical, electron, and photon interactions, whereas energy deposition by ions is avoided.

## 5. Conclusions

The introduction of a mesh with a large open area between the plasma and the substrate offers the exclusion of heavy charged particles such as ions from plasma–surface interactions, while other parameters are maintained. Since ion bombardment during direct plasma exposure is also related to destruction and damaging, for example, recognized by a broadening of FTIR bands, this so-called near-plasma chemistry approach allows a more-defined plasma chemical interaction. Here, the interaction of fast electrons with reactive species (radicals) at the surface might be paramount. The potential of this new methodology has been demonstrated for improving the wettability of PTFE films, as well as for enhanced porosity within SiOx coatings, both at room-temperature conditions. NPC applied to PTFE etching results in attenuated hydrophobic recovery, important for PTFE functionalization and adhesion. Cycles of SiOx deposition, leaving a sacrificial hydrocarbon amount, followed by oxygen etching, removing the residual hydrocarbons, keeps an intact, open Si–O–Si structure under NPC conditions. Hence, high porous volumes have been achieved that are of great interest for diffusion control, gas selection, and as low-refractive index materials that can be deposited on sensitive substrate materials due to the mild plasma conditions operating at room temperature. Here, room-temperature conditions have been studied. In other cases, NPC might also be applied for intentional lowering of the substrate temperature by avoiding strong energy deposition. Further application areas will likely benefit from NPC surface engineering in the future.

## Figures and Tables

**Table 1:** Refractive index and porosity values for samples fabricated using the near-plasma chemistry methodology at the reactor wall of the asymmetric configuration. The maximum obtained is highlighted in bold characters.

HMDSO/O_2_	Power Deposition [W]	Power Etching [W]	Refractive Index/Porosity *
4/40	100	100	1.416/20%
	100	300	1.418/20%
	300	100	1.456/11%
	300	300	1.443/14%
**5/40**	80	100	1.434/16%
	**100**	**100**	**1.405/23%**
	125	100	1.412/21%
6/40	100	100	1.424/18%

* Porosity values are obtained by applying the volume averaging theory (VAT) with an effective medium approximation.

## Data Availability

Data will be made available on request.
